# Synergistic Effect of S224P and N383D Substitutions in the PA of H5N1 Avian Influenza Virus Contributes to Mammalian Adaptation

**DOI:** 10.1038/srep10510

**Published:** 2015-05-22

**Authors:** Jiasheng Song, Jing Xu, Jianzhong Shi, Yanbing Li, Hualan Chen

**Affiliations:** 1 State Key Laboratory of Veterinary Biotechnology, Harbin Veterinary Research Institute, Chinese Academy of Agricultural Sciences, 427 Maduan Street, Harbin 150001, People’s Republic of China

## Abstract

The adaptation of H5N1 avian influenza viruses to human poses a great threat to public health. Previous studies indicate the adaptive mutations in viral polymerase of avian influenza viruses are major contributors in overcoming the host species barrier, with the majority of mammalian adaptive mutations occurring in the PB2 protein. However, the adaptive mutations in the PA protein of the H5N1 avian influenza virus are less defined and poorly understood. In this study, we identified the synergistic effect of the PA/224P + 383D of H5N1 avian influenza viruses and its ability to enhance the pathogenicity and viral replication in a mammalian mouse model. Interestingly, the signature of PA/224P + 383D mainly exists in mammalian isolates of the H5N1 influenza virus and pdmH1N1 influenza virus, providing a potential pathway for the natural adaptation to mammals which imply the effects of natural adaptation to mammals. Notably, the mutation of PA/383D, which is highly conserved in avian influenza viruses, increases the polymerase activity in both avian and human cells, and may have roles in maintaining the avian influenza virus in their avian reservoirs, and jumping species to infect humans.

The highly pathogenic avian influenza (HPAI) H5N1 viruses continue circulation in avian populations results in sporadic infections in humans with a high mortality rate. Human H5N1 infections have a potential for human to human transmissibility^1^,^2^, which raises a great threat to public health. As of October 2014, there have been 668 confirmed cases of H5N1 in humans, resulting in 393 deaths ( http://www.who.int). The adaptive mutations^3^,^4^,^5^ or genetic re-assortments^6^,^7^,^8^ are major contributors for allowing avian influenza viruses to cross the avian species barrier to infect humans.

Previous studies have demonstrated that multiple viral components play a role in pathogenicity and adaptation of the avian influenza virus in mammals. Strains of the human influenza virus preferentially bind α-2,6 sialic acid (SA) while strains of the avian influenza virus bind α-2,3 SA^9^,^10^,^11^. Adaptation of the avian influenza virus could alter the SA receptor-binding specificity to human cell receptor (α2-6 SA) and potentially contribute to human-to-human transmission of H5N1 avian influenza viruses^1^,^2^. Additionally, the presence of a multibasic cleavage site in the hemagglutinin (HA) of highly pathogenic subtype H5 avian viruses also increases virulence both in chicken and mice^4^,^12^. Multiple studies have reported that the NS1 protein of the H5N1 virus is a key component of virulence in mammals^13^,^14^,^15^. Fan, *et al.* demonstrated that two amino acid residues in the matrix protein M1 contribute to differences in virulence of the H5N1 viruses in mice^16^.

The viral polymerase, the nucleoprotein (NP), and the viral RNA genome form the ribonucleoprotein (RNP) complex, which is required for both transcription and viral genome replication^17^. The influenza polymerase is a heterotrimer composed of the subunits PB1, PB2, and PA, and it is widely accepted that adaptive mutations in the influenza polymerase contribute to overcoming species’ barriers^18^. The majority of mammalian adaptive mutations occur in the PB2 protein^3^,^4^,^5^,^19^,^20^,^21^,^22^,^23^,^24^,^25^,^26^. E627K and D701N are two well-characterized mutations of the PB2 protein that are critical for mammalian adaptation of multiple subtypes of the avian influenza viruses^3^,^4^,^5^,^19^,^27^,^28^. However, the accumulative evidence shows the PA protein also plays a critical role in adaptation and pathogenicity in mammals^29^,^30^,^31^,^32^,^33^,^34^. Song, *et al.* reported that a threonine-to-isoleucine substitution at position 97 in the PA protein enhanced the pathogenicity of the H5N2 strain in mice and viral replication in mammalian cells^30^. Mehle, *et al.* reported the substitution of human-origin PA subunits into the avian influenza virus polymerase alleviated restriction in human cells^32^. Zhang, *et al.* reported the PA and NS genes from pdmH1N1 made the H5N1 virus transmissible through respiratory droplets in guinea pigs^6^. It has been reported that the avian H9N2 reassortants with the PA subunit from pdmH1N1can significantly increase the pathogenicity in mice^29^. Finally, Fan, *et al.* found the amino acid residue at position 185 of PA could affect the pathogenicity in mice for avian H5N1 viruses^33^.

We previously reported two genetically similar H5N1 AIVs, A/duck/Hubei/49/05 (R-DK/49) and A/goose/Hubei/65/05 (R-GS/65), that showed significant difference in their virulence in ducks^35^,^36^. The two viruses only differ by 20 amino acids in their genome, including three amino acids at positions 204, 224, and 383 in PA, two amino acids at positions 237 and 738 in PB1, four amino acids at positions 61, 220, 292, and 389 in PB2, three amino acids at positions 10, 102, and 521 in HA, three amino acids at positions 38, 52, and 362 in NA, two amino acids at positions 144 and 156 in M1, and three amino acids at positions 156, 180, and 204 in NS1^35^. We identified that the substitution of two amino acids, S224P and N383D, in PA contributed to the highly virulent phenotype of H5N1 virus R-DK/49 in ducks^35^. In this study, we found that the R-DK/49 and R-GS/65 showed differences of virulence in mice by over 6,000 fold [50% mouse lethal dose (MLD_50_), <0.8 versus 4.6 log_10_ 50% egg infectious dose (EID_50_)]. We investigated the effects of mutations of S224P and N383D in the PA protein of H5N1 influenza viruses on the pathogenicity and viral replication in mice. In addition, most mammalian adaptive mutations in the polymerase of avian influenza virus are correlated with enhanced polymerase activity in mammalian cells^19^,^20^,^23^,^24^,^30^,^37^ so we tested the contribution of residue 224 and 383 to viral polymerase activity in both human and avian cells. Finally, we analyzed the polymorphisms of the residues PA/224 and PA/383 in natural isolates of influenza viruses.

## Results

### Synergistic effect of residues PA/224 and PA/383 on pathogenicity in mice

**Figure 1:**
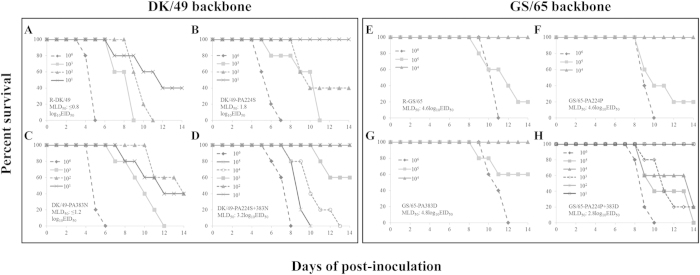
Comparison of pathogenicity of R-DK/49, R-GS/65 and their derived mutants at the residues of 224 and 383 of PA protein in mice. Six-week-old female BALB/c mice (*n = *5/group) were intranasally inoculated with 50 μl of the recombinant avian H5N1 viruses created by reverse genetics and monitored for 14 days for mortality. The MLD_50_ was calculated using the method of Reed and Muench. (A), R-DK/49; (B), DK/49-PA224S; (C), DK/49-PA383N; (D), DK/49-PA224S + 383N; (E), R-GS/65; (F), GS/65-PA224P; (G), GS/65-PA383D; (H), GS/65-PA224P + 383D.

Using plasmid based reverse genetics, we generated a series of viruses mutated at positions 224 and 383 in the PA protein and evaluated their virulence in mice as previously described^13^. GS/65-PA224P + 383D has a MLD_50_ of 2.8log_10_EID_50_ in contrast to MLD_50_ of 4.6log_10_EID_50_ in R-GS/65, indicating the double substitution significantly increases the virulence of the parent virus R-GS/65 in mice (FIG. 1). At the backbone of R-DK/49, the double substitutionP224S + D383N, greatly reduced the virulence with a 100-fold MLD_50_ increase compared with parent virus R-DK/49 in mice (FIG. 1). However, the single substitution of either 224 or 383 had limited effect on the pathogenicity, resulting in little change of the MLD_50_ in both the backbone of R-DK/49 and R-GS/65 (FIG. 1). To determine viral replication and spread, mice (n = 3) were inoculated with a dose of 10^6^ EID_50_ then euthanized on day 3 post-infection (p.i.). The lungs, kidneys, spleens, and brains were collected for viral titration. The R-GS/65 replicated only in the lungs, while its derived mutant GS/65-PA224P + 383D replicated more efficiently in multiple organs (Table 1). There was no significant virus titer reduction observed for the single or double mutants of R-DK/49 at a dose of 10^6^EID_50_ (Table 1). Furthermore, the PA/S224P + N383D substitutions did not convert R-GS/65 to be as pathogenic as R-DK/49. The PA/P224S + D383N substitutions did not completely attenuate the R-DK/49 virus as much as R-GS/65 (FIG. 1 and Table 1). These data support the synergistic effect of residue 224 and 383 in the PA protein on the pathogenesis in mice and suggest other gene segments may also contribute to virulence in mice.

### Polymerase activity of mutant PA proteins in both mammalian and avian cells

**Figure 2:**
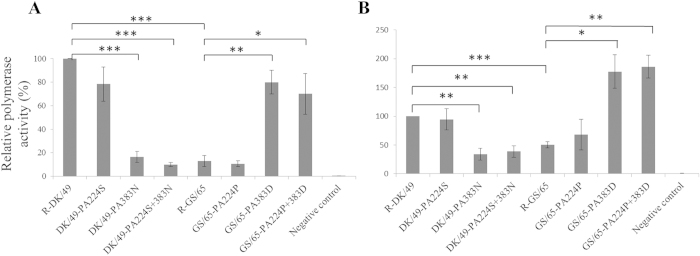
Polymerase activities of R-DK/49 or R-GS/65 with different PA mutations in a minigenome assay. Polymerase complex and NP expression plasmids together with the minigenome firefly luciferase reporter plasmid phumPolI-T-Luc or paviPolI-T-Luc were transfected into 293T cells (A) or DF1 (B) cells, respectively. Data are expressed as the mean ± standard deviation (SD) for three independent experiments and are standardized to the activity of R-DK/49 (100%). Statistical significance was determined using the unpaired Student’s *t*-test with two-tailed analysis (GraphPad Software, La Jolla, CA). *p < 0.01, **p < 0.001, ***p < 0.0001. Negative control, transfection of firefly luciferase reporter only.

Here we examined the effect of the PA/S224P and PA/N383D substitutions on polymerase activity in both human and avian cells. The polymerase activity was normalized against R-DK/49 (set as 100%). In 293T cells, the polymerase activity of mutant DK/49-PA383N was reduced to 16% (p < 0.0001, n = 3) (FIG. 2A). Similarly, a significant reduction in polymerase activity was observed for DK/49-PA224S + 383N (10%; p < 0.0001, n = 3) (FIG. 2A). The polymerase activity of R-GS/65 is low compared to R-DK/49(13% versus 100%; p < 0.0001, n = 3) (FIG. 2A). At the backbone of GS/65, we found the single mutation PA/383D and the double mutation PA/224P + 383D enhanced the viral polymerase activity by 5-6 folds (FIG. 2A). There was no significant change of polymerase activity found in the mutants substituted at residue 224, indicating the existence of unknown mechanisms involved in virulence (FIG. 2). Interestingly, a similar pattern of change in polymerase activity was observed in DF1 cells. At the backbone of DK/49, we found the single mutation PA/383N and the double mutation PA/224S + 383N reduced the polymerase activity by 3 folds (FIG. 2B). On the contrary, the single mutation PA/383D and the double mutation PA/224P + 383D increased the polymerase activity of GS/65 by 3-4 folds (FIG. 2B). The exchange of the PA/383 residue significantly altered the polymerase activity in both avian and human cells at the backbone of GS/65 or DK/49 using the mini-genome assay (FIG. 2).

### Polymorphisms at residues 224 and 383 of the PA protein of influenza viruses

**Figure 3:**
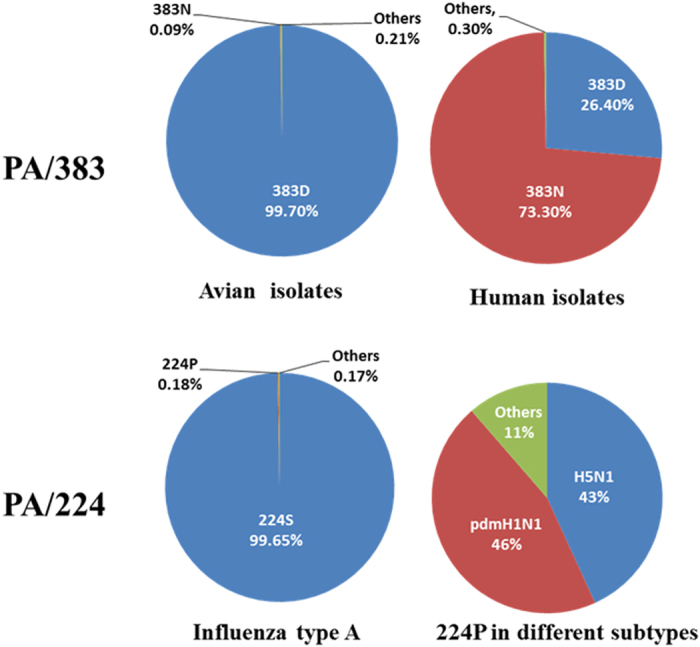
Polymorphism of the residue 383 and the residue 224 in the PA protein of influenza A viruses. A total of 10524 PA sequences from avian isolates (all subtypes) and a total of 7252 PA sequences from human isolates (H1, H2, H3 subtypes, excluding pdmH1N1) were aligned for analyzing the polymorphism of PA 383 residue in avian species and humans. Additionally, all the available PA sequences (a total of 24981) of influenza A viruses were aligned the polymorphism of PA 224 residue. Highly pathogenic signature PA/224P mostly exists in H5N1 and pdmH1N1 subtypes of influenza viruses. Sequence search and alignment were performed by the online service of the Influenza Research Database ( http://www.fludb.org), in October 2014.

To determine the residue changes at position 383 in the PA protein, we aligned a total of 10524 PA sequences of influenza viruses from avian isolates and 7252 PA sequences from human isolates (H1, H2, H3 subtype, excluding pdmH1N1). We found PA/383D is dominant (99.7%) with only 0.09% (9/10542) of strains harboring the mutation PA/383N in avian influenza viruses. Surprisingly, PA/383D only took up 26.4% and the residue PA/383N took up 73.3% in human influenza viruses (FIG. 3). This result may indicate the residue PA/383D is essential for maintaining the influenza virus in avian reservoirs but not in humans. The mutation PA/224S is dominant and the PA/224P is rare in the all influenza A type viruses (FIG. 3). The majority of the mutation PA/224P exists only in H5N1 viruses and the triple re-assortment pdmH1N1 influenza viruses (FIG. 3). Here we found the acquisition of double mutations PA/224P + 383D significantly increased viral pathogenicity and viral replication both in mice and domestic ducks^35^. In nature, only 0.5% of H5N1 avian isolates contain the double mutations PA/224P + 383D, but there is a significant increase rate 3.4% in H5N1 mammalian isolates bearing the PA/224P + 383D. These results may indicate that the highly pathogenic signature PA/224P + 383D of H5N1 avian influenza viruses may not benefit virus maintenance in avian reservoirs, leading to low occurrence rate in avian isolates. However, the signature of PA/224P + 383D may be beneficial for the mammalian adaptation of the H5N1 avian influenza virus. More importantly, some strains of pdmH1N1including early isolates acquired the pathogenic signature of PA/224P + 383D (FIG. 3 and Supplemental Table 1); however, the effects of PA/224P + 383D in pdmH1N1 still need further investigations.

## Discussion

Our previous study demonstrated that mutations at the residue 224 and 383 in the PA protein greatly contributed to the virulence of H5N1 avian influenza in domestic ducks^35^, which is one of avian influenza virus’s natural reservoirs. In this study, we identified mutations at both position 224 and 383 in the PA protein significantly altered the pathogenicity and viral replication of avian H5N1 viruses in mice. Furthermore, we determined the change in amino acid at the residue PA/383 significantly altered the polymerase activity in both mammalian and avian cells, and is consistent with the pathogenicity studies in mice and domestic ducks^35^. The effect of PA/224P + 383D mutations in both mammals and avian reservoirs has been identified. This effect varies from other mammalian adaptive mutations in viral polymerase such as 627K^38^,^39^,^40^, 701N^19^, 271A^23^ in the PB2 protein, which has a limited effect on pathogenicity, viral replication and/or polymerase activity in avian hosts or avian cells.

Intriguingly, PA/383D is highly conserved in avian isolates (99.7%) but not in human isolates (26.4%) of the influenza viruses. The PA/383D mutation is associated with an increased polymerase activity in both avian and human cells. The PA/383D mutation may play an important role in maintaining the avian influenza viruses in avian reservoirs and be a key component for adaptation to humans. The mutation of residue PA/383 is not sufficient for altering viral pathogenicity in mice or ducks and requires the additional mutation of residue PA/224. The synergistic effect of S224P and N383D substitutions in the PA of H5N1 avian influenza on viral pathogenicity and replication was successfully demonstrated in a mouse model, but the underlying mechanisms remain unknown and further investigation is needed. In summary, we identified that the mutations at position of 224 and 383 in the PA protein significantly altered the pathogenicity and viral replication of H5N1 viruses in mice, and the single mutation at residue PA/383 can greatly affect viral polymerase activity in both avian and mammalian cells.

## Materials and Methods

### Facility

Studies with highly pathogenic H5N1 avian influenza viruses were conducted in a biosecurity level 3 laboratory approved by the Chinese Ministry of Agriculture. All animal studies were approved by the Review Board of Harbin Veterinary Research Institute, Chinese Academy of Agricultural Sciences. The study was carried out in strict accordance with the recommendation in the Guide for the Care and Use of Laboratory Animals.

### Cells and plasmids

Human embryonic kidney cells (293T) and chicken embryo fibroblasts cells (DF1) were grown in Dulbecco’s modified Eagle’s medium supplemented with 10% fetal bovine serum plus Penicillin-Streptomycin (100 U/mL) The construction of plasmids for virus rescue and polymerase activity was performed as described previously^35^.

### Generation of reverse genetic viruses

The parental viruses and their derived mutant viruses were rescued as previously described^35^. Briefly, 293T cells were transfected with each of the eight plasmids for influenza viral genome. After 48 h, the supernatant was harvested and injected into embryonated eggs for virus propagation. The rescued viruses were detected by a hemagglutination assay, and the RNA was extracted and analyzed by reverse transcription-PCR (RT-PCR). Each viral segment and mutation was confirmed by sequencing.

### Mouse experiments

Groups of 6-week-old female BALB/c mice (Beijing Experimental Animal Center) were anesthetized with CO2 and inoculated intranasally with the indicated dose of H5N1 influenza virus (50 μl). Mice (n = 3) (10^6^ EID_50_) were euthanized on day 3 p.i. Organs were collected and titrated for virus infectivity in eggs as described previously^3^. The remaining mice (n = 5/group) were monitored for 14 days for mortality. The MLD_50_ was determined by inoculating mice (n = 5) with 10-fold serial dilutions of the virus (50 μl) and calculated by the Reed and Muench method^41^.

### Polymerase activity

293T or DF1cells were transfected with phumPolIT-Luc or paviPolIT-Luc along with the pTK-RL (Promega) and pcDNA3.1 + plasmid constructs expressing the polymerase PB2, PB1, PA, and NP genes (0.5 μg each) plus Lipofectamine 2000 (Invitrogen) as recommended by the manufacturer. Renilla luciferase expressed by pTK-RL was used as an internal control to normalize transfection efficiency. Cell extracts were harvested 30 h post-transfection, and luciferase activity was assayed by using the luciferase assay system (Promega).The assay was standardized against the Renilla luciferase activity. All experiments were performed in triplicate.

### Statistical analysis

Data are expressed as mean ± standard deviation (SD). Statistical significance was determined using unpaired Student’s t-test with two-tailed analysis (GraphPad Software, La Jolla, CA). Significance is defined as *p* < 0.01 and indicated with an asterisk (*).

## Author Contributions

H.C. and J.S. designed the study and wrote the manuscript. J.S., J.X., JZ.S. and Y.L. performed the experiments. All authors reviewed the manuscript.

## Additional Information

**How to cite this article**: Song, J. *et al.* Synergistic Effect of S224P and N383D Substitutions in the PA of H5N1 Avian Influenza Virus Contributes to Mammalian Adaptation. *Sci. Rep.* **5**, 10510; doi: 10.1038/srep10510 (2015).

## Supplementary Material

Supplementary Information

## Figures and Tables

**Table 1:** Replication of H5N1 avian influenza viruses in mice.

**Virus**	**Virus replication on day 3 p.i. (log**_**10**_**EID**_**50**_**/ml ± SD)**			
	**Lung**	**Spleen**	**Kidney**	**Brain**
R-DK/49	7.7 ± 0.1	3.6 ± 0.1	2.8 ± 0.6	3.1 ± 1.2
DK/49-PA224S	7.4 ± 0.1	4.8 ± 0.0	3.3 ± 0.5	2.8 ± 0.4
DK/49-PA383N	7.4 ± 0.1	4.3 ± 0.7	3.5 ± 1.1	3.2 ± 1.3
DK/49-PA224S + 383N	7.5 ± 0.3	3.6 ± 0.1	2.3 ± 0.4	1.4 ± 0.8
R-GS/65	5.0 ± 0.4	<	<	<
GS/65-PA224P	4.9 ± 0.3	1.8 ± 0.6	1.5 ± 1.4	1.0 ± 0.9
GS/65-PA383D	5.3 ± 0.1	0.8 ± 1.3	<	1.3 ± 0.5
GS/65-PA224P + 383D	6.8 ± 0.4	3.1 ± 1.0	2.3±1.4	2.5 ± 1.1

<, Not detectable.
