# Supplementary material for: Synergistic Effect of S224P and N383D Substitutions in the PA of H5N1 Avian Influenza Virus Contributes to Mammalian Adaptation
Source: Sci Rep. 2015 May 22;5:10510. doi: 10.1038/srep10510 (PMC4441148; doi:10.1038/srep10510)
Supplement: Supplementary Information [file srep10510-s1.pdf]

Synergistic Effect of S224P and N383D Substitutions in the PA of H5N1 Avian Influenza Virus Contributes to Mammalian Adaptation

Jiasheng Song, Jing Xu, Jianzhong Shi, Yanbing Li, Hualan Chen*

State Key Laboratory of Veterinary Biotechnology, Harbin Veterinary Research Institute, Chinese Academy of Agricultural Sciences, 427 Maduan Street, Harbin 150001, People's Republic of China

***Correspondence:** Mailing address: Harbin Veterinary Research Institute, CAAS, 427 Maduan Street, Harbin 150001, People's Republic of China. Phone: 86-451-51997168. Fax: 86-451-51997166. E-mail: chenhualan@caas.cn.

Supplemental Table 1. The natural isolates carrying the PA/224P+383D double mutations in the H5N1 and pdmH1N1 influenza A viruses [a]

Virus strains	GenBank accession number	Subtype	Host [b]	627 /PB2	701 /PB2	590-591 /PB2
A/chicken/Anhui/39/2004	ADG59392.1	H5N1	Avian	E	D	GQ
A/chicken/Gansu/44/2004	G59385.1	H5N1	Avian	E	E	GQ
A/swine/Banten/UT3062/2005	ADI46298.1	H5N1	**Swine**	E	D	GQ
A/swine/Banten/UT3063/2005	ADI46299.1	H5N1	**Swine**	E	D	GQ
A/swine/Banten/UT2071/2005	ADI46297.1	H5N1	**Swine**	E	D	GQ
A/swine/Banten/UT3081/2005	ADI46300.1	H5N1	**Swine**	E	D	GQ
A/duck/Hubei/49/2005	ADG59383.1	H5N1	Avian	E	D	GQ
A/goose/Shantou/18442/2005	ABL08901.1	H5N1	Avian	E	D	GQ
A/duck/Vietnam/NCVD06/2005	ACI16726.1	H5N1	Avian	E	D	GQ
A/duck/Guiyang/3996/2005	ABL08960.1	H5N1	Avian	E	D	GQ
A/Shanghai/1/2006	AEO89020.1	H5N1	**Human**	E	D	GQ
A/Indonesia/CDC624/2006	ABI36341.1	H5N1	**Human**	E	D	GQ
A/Indonesia/CDC623/2006	ABI36319.1	H5N1	**Human**	E	D	GQ
A/Indonesia/569H/2006	ABW06126.1	H5N1	**Human**	E	D	GQ
A/great_egret/Hong_Kong/807/2008	ACJ26329.1	H5N1	Avian	E	D	GQ
A/Cambodia/V0401301/2011	AEN68823.1	H5N1	**Human**	E	D	GQ
A/Cambodia/V0606311/2011	AEN68825.1	H5N1	**Human**	K	D	GQ
A/muscovy_duck/Vietnam/OIE_559/2011	BAK39621.1	H5N1	Avian	E	D	GQ
A/chicken/Bangladesh/12VIR_7140_16/2012	AGG53006.1	H5N1	Avian	E	D	GQ
A/California/04/2009	ACP44156.1	pdmH1N1	Human	E	D	SR
A/California/07/2009	ACP44188.1	pdmH1N1	Human	E	D	SR
A/California/08/2009	ACQ63248.1	pdmH1N1	Human	E	D	SR
A/Fukuoka-C/1/2009	ACS69018.1	pdmH1N1	Human	E	D	SR
A/Fukuoka-C/2/2009	ACS92576.1	pdmH1N1	Human	E	D	SR
A/Fukuoka-C/3/2009	S92586.1	pdmH1N1	Human	E	D	SR
A/Kagoshima/1/2009	ACT21990.1	pdmH1N1	Human	E	D	SR
A/Scotland/Glasgow_420755/2009	AFK14748.1	pdmH1N1	Human	E	D	SR
A/Scotland/Glasgow_422823/2009	AFK14763.1	pdmH1N1	Human	E	D	SR
A/Brownsville/31H/2009	ACZ96926.1	pdmH1N1	Human	E	D	SR
A/Brownsville/31OS/2009	ACZ96916.1	pdmH1N1	Human	E	D	SR
A/England/201/2009	AEW10813.1	pdmH1N1	Human	E	D	SR
A/Pensacola/INS213/2009	ADK32457.1	pdmH1N1	Human	E	D	SR
A/Canada/GFA0402/2009	ADJ37270.1	pdmH1N1	Human	E	D	SR
A/Mexico/InDRE3740/2011	AFJ05633.1	pdmH1N1	Human	E	D	SR
A/Helsinki/490/2013	AGV75282.1	pdmH1N1	Human	E	D	SR

A/Helsinki/664/2013	AGV74974.1	pdmH1N1	Human	E	D	SR
A/Helsinki/742/2013	AGV74853.1	pdmH1N1	Human	E	D	SR
A/Helsinki/753/2013	AGV74842.1	pdmH1N1	Human	E	D	SR
A/Helsinki/979/2013	AGV74556.1	pdmH1N1	Human	E	D	SR

[a] Sequence search and alignment were performed by the online service of the Influenza Research Database (http://www.fludb.org), in October 2014.
[b] Boldface type indicates the mammalian infection of the H5N1 influenza virus.
